# Calcific Tendonitis of the Longus Colli Muscle: A Noninfectious Cause of Retropharyngeal Fluid Collection

**DOI:** 10.1155/2014/286190

**Published:** 2014-11-24

**Authors:** Ronak Rahmanian, Chris Diamond

**Affiliations:** Division of Otolaryngology, Head & Neck Surgery, University of British Columbia, Gordon & Leslie Diamond Health Care Centre, 4th Floor, 2775 Laurel Street, Vancouver, BC, Canada V5Z 1M9

## Abstract

Calcific tendonitis of the longus colli (CTLC) muscle is an underrecognized cause of spontaneous acute or subacute neck pain, dysphagia, or odynophagia. Imaging may reveal a retropharyngeal fluid collection leading to the presumed diagnosis of retropharyngeal abscess. Recognition of this uncommon presentation is important to prevent unnecessary surgical incision and drainage. A 44-year-old otherwise healthy male presented with a 2-week history of progressive neck pain, stiffness, and odynophagia. A noncontrast CT scan of the cervical spine revealed a retropharyngeal fluid collection with a small area of calcification anterior to C2. There was a presumed diagnosis of retropharyngeal abscess. The patient was afebrile with normal vital signs. Flexible nasolaryngoscopy was unremarkable. C-reactive protein was elevated but all other bloodwork was normal with no evidence of an infective process. A CT scan was repeated with IV contrast showing no enhancement around the fluid collection. A diagnosis of CTLC was made. The patient was successfully managed with a short course of intravenous steroids and oral NSAIDs with complete resolution of symptoms. Clinically CTLC can mimic more serious disease processes. Identifying pathognomonic imaging findings often confirms the diagnosis. Awareness of this condition by the otolaryngologist will ensure proper patient management and avoidance of unnecessary procedures.

## 1. Introduction

Calcific tendinitis of the longus colli (CTLC) muscle is an underrecognized cause of spontaneous acute or subacute neck pain, dysphagia, or odynophagia. Imaging may reveal a retropharyngeal fluid collection leading to otolaryngology referral for presumed retropharyngeal abscess. Recognition of this uncommon presentation is important to prevent unnecessary surgical incision and drainage. This presentation has received little attention in the otolaryngology literature despite presenting symptoms and findings relevant to the head and neck surgeon. Awareness of CTLC and its presentation is valuable to the practicing otolaryngologist.

## 2. Case Report

A 44-year-old otherwise healthy male presented to the local emergency department on four different occasions over a two-week period with complaints of acute onset, progressive posterior neck pain, and stiffness. On the fourth visit, a two-day history of severe pain causing limited neck mobility and associated odynophagia was described. The patient initially denied any trauma, but in retrospect recalled sustaining a seemingly insignificant fall at work approximately one week prior to the onset of initial symptoms.

**Figure 1:**
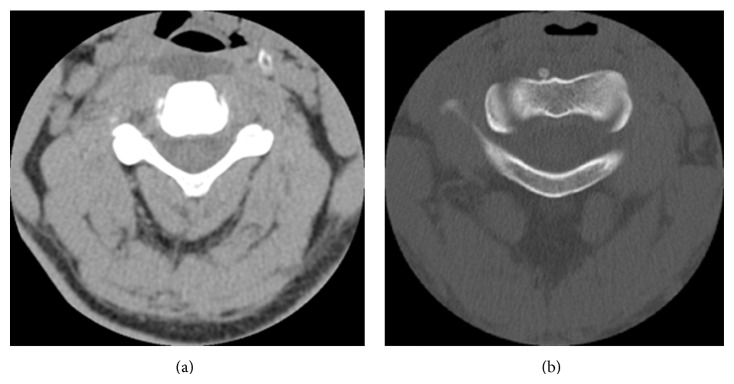
Axial noncontrast CT scan of the cervical spine demonstrating a retropharyngeal fluid collection at the level of C4 (a) and a calcification within the longus colli muscle anterior to C2 (b).

A noncontrast CT scan of the cervical spine was performed in the emergency department revealing a retropharyngeal fluid collection extending from C2 to C6 over a distance of approximately 6 cm, measuring 1 cm in thickness and 2.5 cm in transverse dimension. A small area of calcification was identified at the superior margin of this fluid collection, anterior to C2 (Figure 1). The otolaryngology service was consulted with the presumed diagnosis of retropharyngeal abscess.

**Figure 2:**
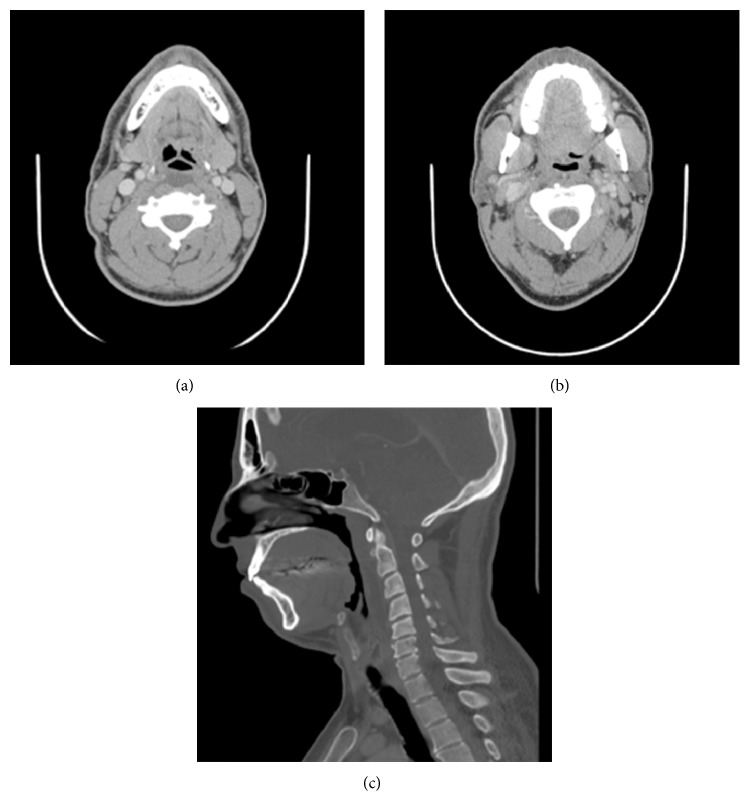
CT scan of the neck with contrast (axial and sagittal views) demonstrating a retropharyngeal fluid collection with no rim enhancement extending from C2 to C6 ((a) and (c)) and a 5 mm focus of calcification within the longus colli muscle anterior to C2 (b) shortly after initial presentation and prior to treatment.

On physical examination, the patient was afebrile with normal vital signs and in no obvious distress, except upon neck movement. Flexible nasolaryngoscopy was unremarkable, with no evidence of abnormal protrusion or fullness of the posterior pharyngeal wall. The C-reactive protein level was elevated at 72.4. The white blood cell count and all other bloodwork were normal, with no evidence of an infective process. A CT scan of the neck was repeated with IV contrast showing no enhancement around the fluid collection and a 5 mm focus of calcification within the longus colli muscle anterior to C2 (Figure 2).

Based on the clinical picture and pathognomonic CT scan findings, a diagnosis of calcific tendinitis of the longus colli (CTLC) muscle was made. The patient was admitted to hospital to complete his workup and manage his discomfort. He was managed successfully with medical therapy, including 3 doses of intravenous steroid followed by a short course of oral NSAIDs. An initial dose of antibiotic was administered until it was determined that an infectious cause was less likely.

**Figure 3:**
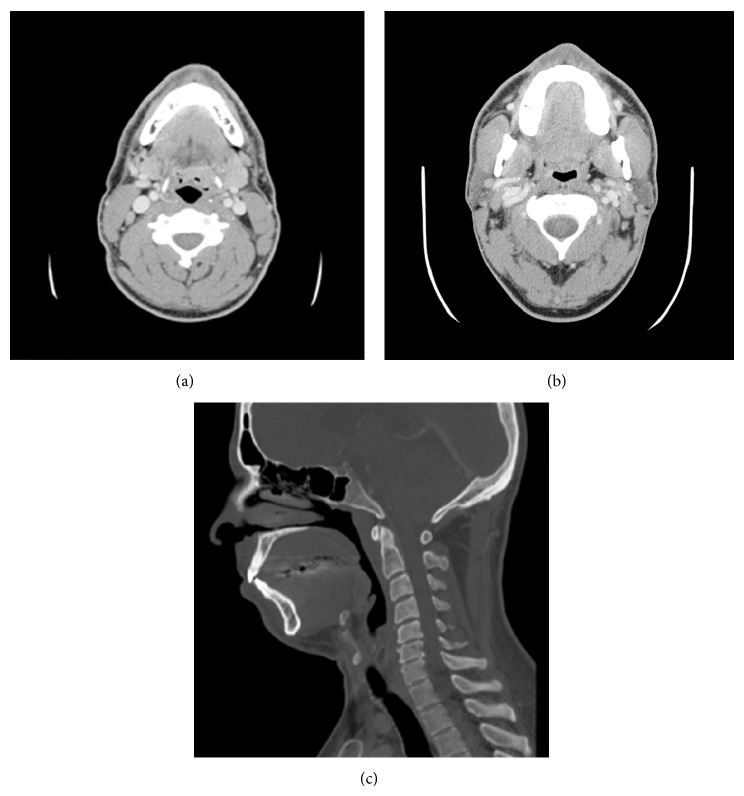
CT scan of the neck with contrast (axial and sagittal views) demonstrating resolution of the retropharyngeal fluid collection ((a) and (c)) and disappearance of the calcification within the longus colli muscle anterior to the C2 cervical vertebra (b) following medical management.

Significant clinical improvement was observed in 2 days, with complete resolution of symptoms and full neck range of motion at 4 weeks. A follow-up CT scan of the neck with contrast revealed complete resolution of the retropharyngeal fluid collection and calcification (Figure 3). Unnecessary surgical incision and drainage was avoided.

## 3. Discussion

Calcific tendinitis of the longus colli (CTLC), also known as retropharyngeal calcific tendinitis, is a relatively rare clinical entity most commonly affecting patients during the third to sixth decades of life. It was first described by Hartley in 1964 [1, 2]. The proposed pathogenesis involves the deposition and rupturing of calcium hydroxyapatite crystals in the longus colli muscle. This leads to induction of a local acute inflammatory response and, in some cases, collection of inflammatory fluid within the retropharyngeal space [3, 4]. The exact etiology of calcium hydroxyapatite deposition is unclear, although previous reports suggest trauma, upper respiratory tract infection, or repetitive movement may be contributing factors [2–5].

The longus colli muscle consists of three portions: superior oblique, vertical, and inferior oblique. It extends from the anterior arch of C1 to the anterior tubercle of T3 and is separated from the retropharyngeal space by the alar and prevertebral layers of the deep cervical fascia [5]. The superior oblique fibers are most vulnerable to calcific deposits [2]. The function of this muscle includes anterior and lateral flexion and rotation of the neck. Local inflammation leads to presentation with the classic triad of neck pain, stiff neck, and odynophagia (with occasional dysphagia) [2, 6, 7]. In the presence of these nonspecific symptoms, important and more dangerous diagnoses such as retropharyngeal abscess/infection, extradural hemorrhage, cervical disc herniation, vertebral body trauma, cervical osteomyelitis, or meningitis should be ruled out [2, 5].

The typical presentation includes a history of acute onset, progressive neck pain with limited range of motion and often associated odynophagia or dysphagia. Nasolaryngoscopy may reveal some edema of the posterior pharyngeal wall, but may be normal. Laboratory testing may demonstrate mild leukocytosis and raised inflammatory markers [1, 2, 5–10]. Pathognomonic radiologic findings are used to confirm the diagnosis. A CT scan with IV contrast typically demonstrates calcium deposits anterior to the body of C2 with associated soft tissue edema or a fluid collection with no rim enhancement in the prevertebral or retropharyngeal space [2, 5].

CTLC is a self-limiting clinical entity with expected spontaneous resolution over a 1-to-2-week period. Treatment involves symptomatic support with analgesia, anti-inflammatory medicine and avoidance of aggravating neck movements [2–5, 11]. Surgical incision and drainage should be avoided.

## 4. Conclusion

Clinically CTLC can mimic more serious disease processes. Identifying pathognomonic findings on imaging often confirms the diagnosis. Recognition and awareness of this condition by the otolaryngologist will ensure proper patient management and avoidance of unnecessary procedures.
